# Colony Fingerprint-Based Discrimination of *Staphylococcus* species with Machine Learning Approaches

**DOI:** 10.3390/s18092789

**Published:** 2018-08-24

**Authors:** Yoshiaki Maeda, Yui Sugiyama, Atsushi Kogiso, Tae-Kyu Lim, Manabu Harada, Tomoko Yoshino, Tadashi Matsunaga, Tsuyoshi Tanaka

**Affiliations:** 1 Division of Biotechnology and Life Science, Institute of Engineering, Tokyo University of Agriculture and Technology, 2-24-16, Naka-cho, Koganei, Tokyo 184-8588, Japan; y_maeda@cc.tuat.ac.jp (Y.M.); y-sugiyama@st.go.tuat.ac.jp (Y.S.); akogiso9305@gmail.com (A.K.); y-tomoko@cc.tuat.ac.jp (T.Y.), tmatsuna@cc.tuat.ac.jp (T.M.); 2 Malcom Co., Ltd., 4-15-10, Honmachi, Shibuya-ku, Tokyo 151-0071, Japan; lim@malcom.co.jp (T.-K.L.); harada@malcom.co.jp (M.H.); 3 Waseda Research Institute for Science and Engineering, Waseda University, 3-4-1 Okubo, Shinjuku-ku, Tokyo 169-8555, Japan

**Keywords:** colony fingerprinting, *Staphylococcus* species, machine learning, lens-less imaging

## Abstract

Detection and discrimination of bacteria are crucial in a wide range of industries, including clinical testing, and food and beverage production. *Staphylococcus* species cause various diseases, and are frequently detected in clinical specimens and food products. In particular, *S. aureus* is well known to be the most pathogenic species. Conventional phenotypic and genotypic methods for discrimination of *Staphylococcus* spp. are time-consuming and labor-intensive. To address this issue, in the present study, we applied a novel discrimination methodology called colony fingerprinting. Colony fingerprinting discriminates bacterial species based on the multivariate analysis of the images of microcolonies (referred to as colony fingerprints) with a size of up to 250 μm in diameter. The colony fingerprints were obtained via a lens-less imaging system. Profiling of the colony fingerprints of five *Staphylococcus* spp. (*S. aureus*, *S. epidermidis*, *S. haemolyticus*, *S. saprophyticus*, and *S. simulans*) revealed that the central regions of the colony fingerprints showed species-specific patterns. We developed 14 discriminative parameters, some of which highlight the features of the central regions, and analyzed them by several machine learning approaches. As a result, artificial neural network (ANN), support vector machine (SVM), and random forest (RF) showed high performance for discrimination of theses bacteria. Bacterial discrimination by colony fingerprinting can be performed within 11 h, on average, and therefore can cut discrimination time in half compared to conventional methods. Moreover, we also successfully demonstrated discrimination of *S. aureus* in a mixed culture with *Pseudomonas aeruginosa*. These results suggest that colony fingerprinting is useful for discrimination of *Staphylococcus* spp.

## 1. Introduction

*Staphylococcus* species are widely distributed in various animals including human and livestock, and are frequently isolated as common pathogens from clinical specimens. The skin, anterior nares, and mucous membranes of human and other animals including mammals and birds contain *Staphylococcus* spp., but hosts can vary depending on the bacterial species [1]. *S. aureus* is one of the most pathogenic staphylococci that causes a range of severe infections such as suppurative diseases and toxic-shock syndrome, while other staphylococci including *S. epidermidis* and *S. saprophyticus* can also be pathogenic to humans. In particular, multi-drug resistant *Staphylococcus* spp. such as methicillin-resistant *S. aureus* (MRSA), vancomycin-resistant *S. aureus* (VRSA), and methicillin-resistant *S. epidermidis* (MRSE) give rise to hospital-acquired infection. *S. aureus* is also recognized as a common food-borne pathogen that produces protein exotoxins called staphylococcal enterotoxins (SEs). Various types of SEs have been identified and showed emetic activity. For example, milk or milk-based products (e.g., cheese and yogurt) frequently cause *S. aureus* outbreaks [2]. It was reported that raw cow’s milk usually contains 100–200 colony formation unit (cfu)/mL of *S. aureus*, which can reach 10^4^ cfu/mL when contaminated [3,4,5]. The detected *S. aureus* can retain the SE production ability. Therefore, insufficient temperature control during the production process can readily lead to bacterial growth and SE accumulation in milk-based products. Thorough suppression of bacterial growth and its evaluation throughout the production lines are significantly important for food safety. Consequently, simple, rapid, and inexpensive methods for detection and discrimination of *Staphylococcus* spp., including *S. aureus*, are highly required.

For bacterial detection and discrimination, a number of methods based on phenotypic (e.g., specific enzymatic activities) and genotypic (e.g., sequence of 16S ribosomal DNA) analyses have been proposed. Particularly for *Staphylococcus* spp., traditional phenotypic analyses, i.e., mannitol salt agar test and coagulase test, have been employed to distinguish *S. aureus* from other species. *Staphylococcus* spp. can selectively grow on mannitol salt agar containing high NaCl (~7.5%), D-mannitol, and phenol red [6]. Among *Staphylococcus* spp., *S. aureus* is a representative species that ferments mannitol. The resulting proton decreases the pH of the mannitol salt agar around the *S. aureus* colonies, and the phenol red in the agar medium indicates the pH change. The coagulase test utilizes coagulase activity, which converts fibrinogen to fibrin and induces clotting of plasma, of *S. aureus* [7]. Both methods have been widely employed. However, substantial number of bacterial cells is required for discrimination, and thus each test takes 24–48 h.

Recently, novel methods for bacterial discrimination have been proposed. For example, matrix-assisted laser desorption/ionization time-of-flight mass spectrometry (MALDI-TOF-MS) is a powerful tool for bacterial discrimination and diagnosis [8,9]. Well-grown colonies of interest are picked, followed by direct ionization of the peptides derived from bacterial cells with the aid of the matrix. The obtained peptide-mass fingerprints provide clues to discriminate bacterial species. Raman spectroscopy is also employed for bacterial discrimination [10]. Analysis with Raman spectroscopy provides structural fingerprints of the molecules contained in the bacterial cells. Bhunia et al. has proposed a simple method for bacterial discrimination based on light scattering patterns from colonies (referred to as bacterial rapid detection using optical scatter technology (BARDOT)) [11,12,13,14,15]. In this method, the bacterial colonies grown on a Petri dish were irradiated by a red laser, followed by generation of light scattering patterns behind the dish. The light scattering patterns can vary depending on the three-dimensional (3D) morphology of bacterial colonies, which are highly species-specific. These methods allow highly accurate and high-throughput analyses. However, these methods need millimeter-scale colonies for accurate discrimination, leading to long assay time. Although, with BARDOT approach, miclocolonies (the diameter range of 100–200 mm) were used for discrimination of *Salmonella enterica*, *Listeria monocytogenes*, and *Escherichia coli*, discrimination of bacteria in the identical genus was not attempted [11]. In addition, these methods still require expensive equipment (e.g., laser and computer-controlled actuators for BARDOT), and thus it is difficult to offer affordable bacterial tests.

To address these issues, we have proposed a simple and rapid method for bacterial discrimination called “colony fingerprinting” [16]. Colony fingerprinting is a bacterial discrimination method based on the imaging analysis of bacterial microcolonies with sub-millimeter scale. Colony images termed “colony fingerprints” are acquired with a wide-field imaging system [17,18,19,20,21,22,23], enabling observation at square millimeter-scale view in one shot with high time-resolution. Extraction and multivariate analyses of the image features of the colony fingerprints allowed discrimination of bacterial species. The equipment for lens-less imaging was composed of only blue light emitting diode (LED) and a complementary metal-oxide semiconductor (CMOS) image sensor without optical lens. This means that colony fingerprinting can be carried out using a simple, inexpensive, and compact devise. In our previous study [16], it was demonstrated that five microorganisms belonging to different genera (*E. coli*, *S. aureus*, *Pseudomonas aeruginosa*, *S. enterica*, and *Candida albicans*) could be discriminated based on the colony fingerprinting. On the contrary, discrimination of closely related bacteria in identical genera was not intensively studied. We only demonstrated that two *Staphylococcus* spp. (*S. aureus* and *S. epidermidis*) were distinguishable with linear discrimination analysis (LDA) of their colony fingerprints. In this study, we expanded the discrimination targets to five *Staphylococcus* spp., i.e., *S. aureus*, *S. epidermidis*, *S. haemolyticus*, *S. saprophyticus*, and *S. simulans*, all of which have been isolated from humans, and can be pathogenic. To precisely discriminate these closely related species, we developed novel discriminative parameters, which were not employed in our previous study [16], as image features of colony fingerprints. Furthermore, discrimination of the target bacteria was attempted using several machine learning approaches. We also discuss the relationship between actual colony morphology and colony fingerprints. The results of this study indicate that colony fingerprinting showed considerable promise as a tool for bacterial discrimination even within the closely related species.

## 2. Materials and Methods

### 2.1. Bacterial Strains

*S. aureus* ATCC 6538, *S. epidermidis* ATCC 14990, and *P. aeruginosa* ATCC 9027 were obtained from American type culture collection (ATCC, Manassas, VA, USA). *S. haemolyticus* NBRC 109768, *S. saprophyticus* subsp. *saprophyticus* NBRC 102446, and *S. simulans* NBRC 109714 were obtained from National Institute of Technology and Evaluation (NITE), Biotechnological Resource Center (NBRC, Kisarazu, Chiba, Japan). *Staphylococcus* spp. were cultivated in lysogeny broth (LB) medium (5 mL) with shacking at 37 °C overnight prior to colony formation testing. The cell concentration was determined by cell counting using a microscope and hemocytometer.

### 2.2. Lens-Less Imaging System

Setup of the lens-less imaging system and the observation methods employed in this study were described in our previous study [16]. The lens-less imaging system consists of a CMOS image sensor (2048 × 1536 pixels, pixel size: 3.2 μm, imaging area: 6.55 × 4.92 mm^2^, DFK61BUC02, The Imaging Source Europe GmbH, Bremen, Germany) and a blue light-emitting diode (LED, λ = 465 nm) located 10 cm above the sensor. Bacterial cells were spread on LB-agar embedded in a microchamber composed of a glass slide, cover grass, and spacer seals (9 × 9 mm^2^, thickness 300 μm each). The microchamber without a cover glass was fulfilled with 1.5% (*w*/*v*) agarose-containing LB medium in the liquid state. At this point, a sticky side of the spacer seal was covered by a release film. A cover glass was mounted to make the surface of LB-agar flat. After 20 min, LB-agar was solidified, and then the cover glass and the release film covering the spacer seal were carefully removed. Bacterial suspension (3.2 × 10^5^ cells/mL, 1 μL) was dropped on the LB-agar. A cover glass was mounted on the LB-agar again. The cover glass and spacer seal were tightly agglutinant to prevent water evaporation. The microchamber containing bacterial cells was placed on the CMOS image sensor. The entire system (CMOS sensor, microchanber and LED) were kept in an incubator at 37 °C [16]. Images were automatically captured every 5 min (exposure time: 1/18 s) under the control of the IC Capture 2.2 software (The Imaging Source Europe GmbH, Bremen, Germany).

### 2.3. Imaging Processing

The image analysis described below was performed using ImageJ [24] and MATLAB (The MathWorks, Inc., Natick, Massachusetts, MA, USA). First, the contrast of original lens-less images was enhanced by remapping the data values to fill the entire intensity range (0, 255) using the auto-adjusting function for intensity values in MATLAB. Subsequently, each pixel value is subtracted from the maximal pixel value, and the difference is used as the pixel value in the output images; i.e., black-and-white balance is inverted. Then, the images were binarized based on the Otsu’s thresholding method [25], by which the pixels were represented in 10 gray levels, and dichotomized into two classes (i.e., background and colony regions) with a threshold at the maximum level. After the binarization, fill-up processing was executed to determine colony regions. Finally, discriminative parameters, which are described in detail below, were calculated from the contrast-adjusted and inverted lens-less images.

### 2.4. Discrimination Analysis

In this study, 14 parameters (i.e., growth rate, μ_max_; histogram deviation, G; average intensity, I; half central intensity, I_1/2_; quarter central intensity, I_1/4_; dounutness, D; central dounutness, D_c_; entropy, H; energy, En; energy density, Ed; weighted center difference, W; roundness, R; Zernike moment, Z; and solidity, S) were extracted from the colony images captured with the lens-less imaging system, which are referred to as “colony fingerprints” with a size of approximately 250 μm. Individual parameters were explained in Table S1. The parameters were computed using MATLAB software.

Discrimination analysis was performed using R 3.1.2 (R Foundation for Statistical Computing, Vienna, Austria) [26]. LDA was operated using the function lda. k-nearest neighbor algorithm (k-NN), naive Bayes classifier (NB), artificial neural network (ANN), support vector machine (SVM), and random forest (RF) were operated using the caret package; methods knn, nb, nnet, svmRadial, and rf, respectively. For k-NN, the optimal value of k was 11. Leave-one-out cross-validation was employed to assess the generalizable discrimination accuracy of the models.

## 3. Results

### 3.1. Colony Fingerprints of Staphylococcus spp.

**Figure 1:**
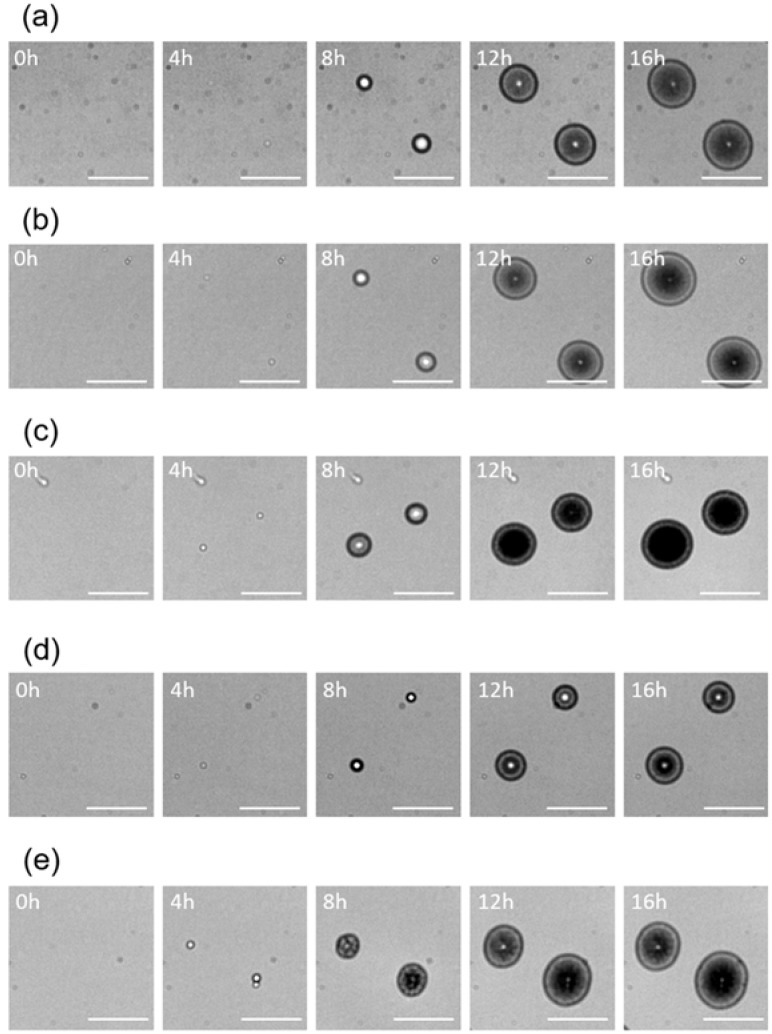
Variation in colony fingerprints of five *Staphylococcus* spp., (**a**) *S. aureus*, (**b**) *S. epidermidis*, (**c**) *S. haemolyticus*, (**d**) *S. saprophyticus*, and (**e**) *S. simulans*, cultivated for 16 h (scale bar = 400 µm).

Using the lens-less imaging system [16], time-lapse images of colony formation of five *Staphylococcus* spp. were obtained. (Figure 1, see also Supplementary Information Figure S1). It should be noted that, in our previous study [16], we already confirmed that colony fingerprints were reproducible even when different operators did the experiments using different culture lots. It was observed that all of the five species generated round-shaped colony fingerprints. In general, a region with high intensity (shown as a central white dot) were surrounded by a region with low intensity (shown in black) at the initial stages of the colony formations (e.g., typically Figure 1a,b,d, at 8 h). The regions with low intensity expanded outward during the growth of colonies, while a white ring was observed on the inner side of the outer edge (e.g., typically Figure 1a–e, at 16 h). Characteristic white dots at the central area were also observed (e.g., typically Figure 1a,b,d,e, at 16 h).

**Figure 2:**
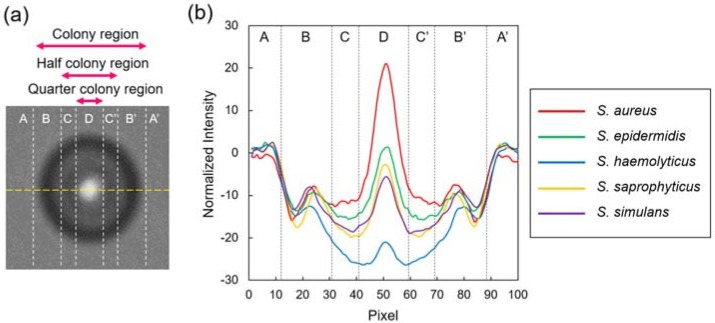
Intensity profiles across the colony fingerprints of *Staphylococcus* spp. (**a**) Intensities were profiled along a yellow line (100 pixel in length) across the colony fingerprints. (**b**) Profiles of the average values of 25 colonies of *S. aureus* (red), *S. epidermidis* (green), *S. haemolyticus* (blue), *S. saprophyticus* (yellow), and *S. simulans* (purple). The intensity values were normalized by subtracting the intensity values at the randomly selected non-colony region. (A and A’) non-colony region, (B–B’) colony region, (C–C’) half colony region, (D) quarter colony region.

Although the general features described above were similar among the five species, distributions of intensity in the colony regions were likely to be species-specific. To quantitatively assess this difference, we selected the colony fingerprints of each species at the size of approximately 250 µm in diameter, and plotted the intensity profiles across the colony fingerprints (Figure 2a). The intensities of the five species at the outer edge region (B and B’ in Figure 2b) were relatively similar. A clear difference appeared at the region with the half diameter of the entire colony region, referred to as half colony region in this study (C–C’ in Figure 2b). Within it, the most central regions with quarter diameter of the entire colony region, referred to as quarter colony region (D in Figure 2b), showed the most variability.

### 3.2. Discriminative Parameters for Colony Fingerprinting of Staphylococcus spp.

In the previous study [16], we discriminated *S. aureus* and *S. epidermidis* by LDA with seven discriminative parameters: Maximum specific growth rate, colony appearance time, relative intensity, histogram deviation, donutness, entropy, and energy density. Among these, colony appearance time and relative intensity could not generate output automatically using the MATLAB software, but required labor-intensive calculations using ImageJ due to the complicated definitions. Our goal was to develop a simple system to discriminate bacteria. Therefore, in the present study, we decided to eliminate these two parameters for discrimination.

We attempted to discriminate 25 colony fingerprints of five *Staphylococcus* spp. (125 colony fingerprints in total) by LDA with the remaining five parameters, i.e., maximal growth rate (μ_max_), histogram deviation (G), donutness (D), entropy (H), and energy density (Ed). These parameters were extracted from the colony fingerprints with a size of approximately 250 μm as described above. It took 11 ± 2 h for the colony fingerprints to reach this size. As a result, discrimination accuracy was 74.4% (Table 1, see also Supplementary Information Table S2). We considered that developing additional discriminative parameters that represent the species-specific features of colony fingerprints was required in order to improve the discrimination accuracy.

Next, we computed the additional nine parameters, i.e., central dounutness (D_c_), energy (En), average intensity (I), half central intensity (I_1/2_), quarter central intensity (I_1/4_), weighted center difference (W), roundness (R), Zernike moment (Z), and solidity (S), all of which can be output automatically using our MATLAB program. Distribution of 14 parameters extracted from the 25 colony fingerprints of five *Staphylococcus* spp. was shown in the Supplementary Information Figure S2. In particular, D, D_c_, I_1/2_, and I_1/4_ strongly highlighted the species-specific features of colony fingerprints because they are determined based on the intensities at the half and quarter colony regions where the intensity much varied species by species (Figure 2b). Subsequently, we examined the 14 parameters extracted from the colony fingerprints of five *Staphylococcus* spp. by analysis of variance (ANOVA, Table S3). Ten parameters including D, D_c_, I_1/2_, and I_1/4_ showed statistically significant differences (*p* < 0.05) among the five *Staphylococcus* spp., while Z, S, W, and R did not. This result would be reasonable since typical colony fingerprints of *Staphylococcus* spp. are radially symmetric without distinct convexity. Although these four parameters might contribute less to discrimination, compared to others, we employed all of the 14 parameters for the following analysis.

**Figure 3:**
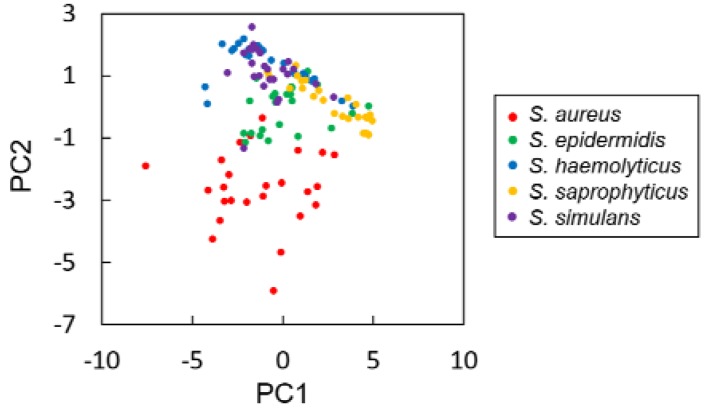
Principal component analysis of the colony fingerprints of five *Staphylococcus* spp., i.e., *S. aureus* (red), *S. epidermidis* (green), *S. haemolyticus* (blue), *S. saprophyticus* (yellow), and *S. simulans* (purple).

Principal component analysis (PCA) was first carried out with the 14 parameters extracted from the colony fingerprints of five *Staphylococcus* spp. PCA revealed that *S. aureus* were relatively separated from four other species (Figure 3). Then, discrimination of the five *Staphylococcus* spp. was performed by LDA with the 14 discriminative parameters. As a result, discrimination accuracy reached 79.2% (Table 1, see also Supplementary Information Table S4). This result indicates that discrimination accuracy was improved by employing additional parameters, but it did not reach 100%. Therefore, we decided to consider other machine learning approaches for better discrimination accuracy.

### 3.3. Comparison of Machine Learning Approaches for Discrimination

As machine learning approaches for colony fingerprinting, we further tested five classifiers, k-NN, NB, ANN, SVM, and RF. With the 14 parameters, discrimination accuracy by k-NN, NB, ANN, SVM, and RF were 80.8%, 83.2%, 99.2%, 98.4%, and 100.0%, respectively (Table 1, see also Supplementary Information Tables S5–S9). When we compared the positive predictive values (PPVs), which reflect the probability that an instance classified into a particular species was actually that species, those of *S. aureus* were equal to or even higher than those of other species with any classifiers, and were 100%, except for the analysis by LDA. This suggests that accurate discrimination of *S. aureus* was relatively easy compared to other species.

### 3.4. Colony Fingerprints of S. aureus in the Presence of Another Bacterium

**Figure 4:**
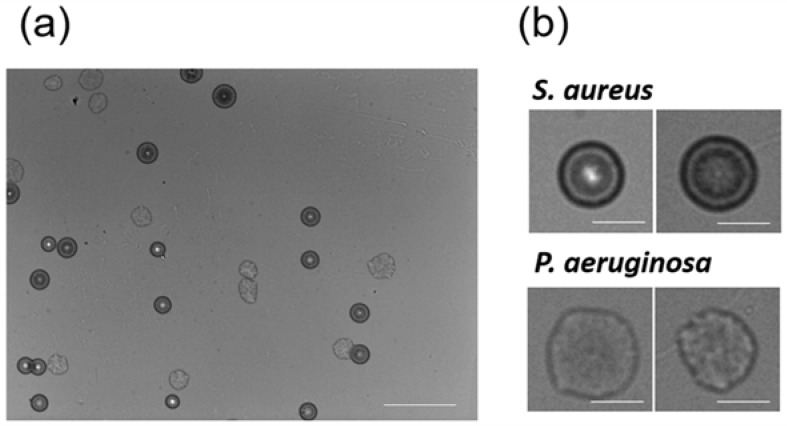
Colony fingerprints of *S. aureus* co-existing with *P. aeruginosa.* (**a**) A wide field of view image of the culture of *S. aureus* and *P. aeruginosa* (scale bar = 1 mm); (**b**) the magnified colony fingerprints of *S. aureus* and *P. aeruginosa* (scale bar = 200 μm).

In the experiments described above, each bacterial species was solely cultivated and visualized. However, actual bacterial contamination can be caused by multiple species. To date, it remained elusive whether co-existence of multiple bacterial species affects the discrimination accuracy of colony fingerprinting. To address this issue, we acquired another 23 colony fingerprints of *S. aureus* in the presence of *P. aeruginosa* (Figure 4). According to our previous study [16], *S. aureus* and *P. aeruginosa* were easily distinguishable. We used the 125 colony fingerprints of the solely cultivated five *Staphylococcus* spp., which were obtained above, as training data, and discriminated those of *S. aureus*, which co-existed with *P. aeruginosa*, as test data by ANN, SVM, and RF. Using SVM, 22 colony fingerprints were correctly classified as *S. aureus*, but one was recognized as *S. epidermidis*. ANN and RF classified all of the 23 colony fingerprints as *S. aureus*. These results suggest that discrimination of *Staphylococcus* spp. with high accuracy based on the colony fingerprinting was successfully demonstrated using ANN, SVM, and RF as classifiers even in the presence of *P. aeruginosa*.

## 4. Discussion

In the present study, we examined the performance of a colony fingerprinting approach [16] for discrimination of five closely related *Staphylococcus* spp. by various machine learning approaches. According to Bergey’s Manual of Systematic Bacteriology (2nd ed.) [1], all of the five species analyzed in this study form smooth, glistening, and raised colonies with entire margin on conventional agar plates. Therefore, we assumed that the morphologies of the colonies growing in the microchambers composed of glass slides [16] could also be similar—resulting in the generation of similar colony fingerprints. The lens-less imaging system allowed us to discriminate these similar colony fingerprints (Figure S1) with a size of up to 250 μm, which could not be discriminated with the bare eye due to the small size. This resulted in rapid discrimination within 11 h on average. We have not evaluated how accurate the colony fingerprinting is if colony fingerprints smaller than 250 μm are used. If smaller colony fingerprints enable accurate discrimination, assay time can be shorter. In addition, the colony fingerprints can be affected by the density populations; colonies in the high colony density area tend to be small in size. This could be caused by the limitation of the nutrition supply from LB-agar medium. In the present study, we did not analyze the colonies located at a too dense area where the colonies merged with other ones. Classification of the merging colonies is the next issue to be solved in the future study.

The lens-less imaging system employed in this study was composed of only blue LED and a CMOS image sensor without any optical lens and actuators. The lens-less imaging systems composed of CMOS image sensor are recently utilized for various cell analyses, in particular for mammal cells. For instance, Bishara et al. analyzed red blood cells with or without malaria parasite infection using a CMOS image sensor and a fiber-optic array [27]. Vercruysse et al. demonstrated the label-free discrimination of leukonocytes (lymphocytes, monocytes and granulocytes) using a CMOS image sensor combined with a microfluidic channel [28]. In these studies, the lens-less imaging systems were developed with super-resolution technologies including image re-construction techniques, because the main observation objects were mammal cells with the size of several micrometers. As compared to these imaging systems, our system was much simpler, and did not require super-resolution for the purpose of bacterial discrimination.

For the discrimination, 14 parameters were extracted from the colony fingerprints (Table S1). Since a clear difference in intensity appeared at the half-and quarter-colony regions (Figure 2), we employed some parameters (e.g., D, D_c_, I_1/2_, and I_1/4_), which reflected such differences. Indeed, ANOVA revealed that such parameters exhibited high F values (i.e., the high variations between groups and/or low variations in groups). It was expected that these parameters could strongly contribute to discrimination of the five *Staphylococcus* spp. The quarter-colony regions of the colony fingerprints almost correspond to the central white dot (Figure 2). The high intensity at the white dot suggests that the light passing through the colony was condensed on the photodiode of the CMOS sensor at this region. As mentioned, the *Staphylococcus* spp. form raised elevated colonies with smooth surfaces [1]. These facts suggest that the colonies might behave like a hemisphere lens. This notion is supported by the studies reporting that bacterial colonies can act as biological microlenses in spite of incomplete transparency [29,30]. Therefore, we assumed that differences of the parameter values between species could be attributable to the difference in colony morphology, which can directly correlate to the light focusing property. The 3D morphology of the staphylococcal microcolonies in the microchamber remained elusive, and it will be analyzed in the near future for example by meads of confocal microscopy [31].

PCA analysis with the 14 parameters of the five *Staphylococcus* spp. revealed another interesting point that *S. aureus* were separated from the other four species (Figure 3). Accordingly, PPVs of *S. aureus* tend to be higher than those of other 4 species (Table 1). *S. aureus* is the most clinically important species among *Staphylococcus* spp. due to the high pathogenicity. The relationship between the high pathogenicity of *S. aureus* and its cell surface property has been well studied [32]. Compared to other *Staphylococcus* spp., *S. aureus* displays various types of host matrix-binding proteins that enable adherence to the matrix-composing proteins including collagen, laminin, elastin, and fibronectin, as well as fibrinogen. This adhesive property leads, in part, to high pathogenicity, and thus distinguishes *S. aureus* from other *Staphylococcus* spp. In addition, it is also well known that *S. aureus* secretes proteins that other *Staphylococcus* spp. do not produce, e.g., coagulase and enterotoxin [1]. Such extracellular components could affect the intercellular adhesion and special arrangement of the cells in the colonies. These could result in variation of the light passing properties of the colonies, leading to generation of the *S. aureus*-specific colony fingerprints.

Nowadays, it is easy to utilize various types of machine learning approaches with open source platforms for statistical analysis like R [26]. We employed k-NN, NB, ANN, SVM, and RF in this study, and found that ANN, SVM, and RF showed high performance. Recently, various bacterial discrimination methods assisted by machine learning approaches were proposed, e.g., light scattering pattern (referred to as BARDOT) [11,12,13,14,15], Raman spectroscopy [33], and MALDI-TOF-MS [34]. In these studies, SVM was the most employed due to the high performance for discrimination as well as ease of implementation with the aid of sophisticated software packages. ANN is a machine learning approach inspired by biological neural networks in the brain. RF is an ensemble method in which multiple decision trees are developed, and final classification is the class with the most votes. The high performance of RF is attributable to high number of decision trees, although discrimination performance of each decision tree is not necessarily high. RF was also employed for bacterial discrimination based on MALDI-TOF-MS [34,35]. These machine learning approaches (ANN, SVM, and RF) can easily deal with nonlinearly separable data, which LDA cannot handle. It remains arguable which ANN, SVM, and RF are the best classifier for colony fingerprinting. The comparison with much larger data set may bring an end to this argument.

In the present study, we demonstrated that these three machine learning approaches were useful for colony fingerprinting. These results suggest that colony fingerprinting has great promise for feasible bacterial tests.

## 5. Conclusions

Discrimination of five species of *Staphylococcus* spp. with colony fingerprinting was successfully demonstrated. We developed novel discriminative parameters that reflect species-specific features of colony fingerprints. As machine learning approaches to analyze these parameters, ANN, SVM and RF showed high performance compared to k-NN, NB, and LDA. By analyzing 14 parameters with RF, discrimination accuracy reached 100%. Furthermore, it was possible to correctly discriminate *S. aureus*, even in the presence of *P. aeruginosa*. This suggests that colony fingerprinting could be useful to analyze contaminated samples with multiple species. It was possible to obtain the colony fingerprints within 11 h on average using the compact and inexpensive equipment. Therefore, colony fingerprinting is a rapid and cost-effective method for detection and discrimination of *Staphylococcus* spp.

## Figures and Tables

**Table 1:** Classification of colony fingerprints of five *Staphylococcus* spp. by machine learning approaches.

Classifier *^a^*	Parameters *^b^*	Accuracy *^c^*	Species	Sensitivity *^d^*	Specificity *^e^*	PPV *^f^*
LDA	μ_max_, G, D, H, Ed	74.4%	*S. aureus*	80.0%	99.0%	95.2%
	(5 parameters)		*S. epidermidis*	72.0%	89.0%	62.1%
			*S. haemolyticus*	64.0%	99.0%	94.1%
			*S. saprophyticus*	80.0%	95.0%	80.0%
			*S. simulans*	76.0%	86.0%	57.6%
LDA	μ_max_, G, I, I_1/2_, I_1/4_, D, Dc, H, En, Ed, W, R, Z, S	79.2%	*S. aureus*	84.0%	99.0%	95.5%
			*S. epidermidis*	76.0%	86.0%	57.6%
	(14 parameters)		*S. haemolyticus*	76.0%	99.0%	95.0%
			*S. saprophyticus*	88.0%	97.0%	88.0%
			*S. simulans*	72.0%	93.0%	72.0%
k-NN	μ_max_, G, I, I_1/2_, I_1/4_, D, Dc, H, En, Ed, W, R, Z, S	80.8%	*S. aureus*	88.0%	100.0%	100.0%
			*S. epidermidis*	84.0%	86.0%	60.0%
	(14 parameters)		*S. haemolyticus*	76.0%	97.0%	86.4%
			*S. saprophyticus*	88.0%	96.0%	84.6%
			*S. simulans*	68.0%	97.0%	85.0%
NB	μ_max_, G, I, I_1/2_, I_1/4_, D, Dc, H, En, Ed, W, R, Z, S	83.2%	*S. aureus*	88.0%	100.0%	100.0%
			*S. epidermidis*	84.0%	91.0%	70.0%
	(14 parameters)		*S. haemolyticus*	76.0%	97.0%	86.4%
			*S. saprophyticus*	88.0%	95.0%	81.5%
			*S. simulans*	80.0%	96.0%	83.3%
ANN	μ_max_, G, I, I_1/2_, I_1/4_, D, Dc, H, En, Ed, W, R, Z, S	99.2%	*S. aureus*	100.0%	100.0%	100.0%
			*S. epidermidis*	100.0%	100.0%	100.0%
	(14 parameters)		*S. haemolyticus*	96.0%	100.0%	100.0%
			*S. saprophyticus*	100.0%	99.0%	96.2%
			*S. simulans*	100.0%	100.0%	100.0%
SVM	μ_max_, G, I, I_1/2_, I_1/4_, D, Dc, H, En, Ed, W, R, Z, S	98.4%	*S. aureus*	100.0%	100.0%	100.0%
			*S. epidermidis*	96.0%	99.0%	96.0%
	(14 parameters)		*S. haemolyticus*	100.0%	100.0%	100.0%
			*S. saprophyticus*	100.0%	100.0%	100.0%
			*S. simulans*	96.0%	99.0%	96.0%
RF	μ_max_, G, I, I_1/2_, I_1/4_, D, Dc, H, En, Ed, W, R, Z, S	100.0%	*S. aureus*	100.0%	100.0%	100.0%
			*S. epidermidis*	100.0%	100.0%	100.0%
	(14 parameters)		*S. haemolyticus*	100.0%	100.0%	100.0%
			*S. saprophyticus*	100.0%	100.0%	100.0%
			*S. simulans*	100.0%	100.0%	100.0%

*^a^* Six types of machine learning approaches, i.e., linear discrimination analysis (LDA), k-nearest neighbor algorithm (k-NN), naive Bayes classifier (NB), artificial neural network (ANN), support vector machine (SVM), and random forest (RF), were employed. *^b^* Up to 14 parameters, i.e., maximum specific growth rate (μ_max_), histogram deviation (G), average intensity (I), half central intensity (I_1/2_), quarter central intensity (I_1/4_), dounutness (D), central dounutness (D_c_), entropy (H), energy (En), energy density (Ed), weighted center difference (W), roundness (R), Zernike moment (Z), and solidity (S), were employed. *^c^* Accuracy = sum of true positive/125 × 100 (%). *^d^* Sensitivity = true positive/25 × 100 (%). *^e^* Specificity = sum of true negative/100 × 100 (%). *^f^* Positive predictive value (PPV) = true positive/sum of colonies predicted as a particular species × 100 (%).

## Supplementary Materials

The following are available online at http://www.mdpi.com/1424-8220/18/9/2789/s1, Table S1: Discriminative parameters used in this study. Figure S1: The colony fingerprints of five *Staphylococcus* spp. acquired in this study. Table S2: Confusion matrix from LDA classification of 125 colony fingerprints based on five parameters. Table S3: ANOVA for the parameters. Figure S2: Distribution of the values of the discriminative parameters extracted from the colony fingerprints of 5 *Staphylococcus* spp. Table S4: Confusion matrix from LDA classification of 125 colony fingerprints based on 14 parameters. Table S5: Confusion matrix from k-NN classification of 125 colony fingerprints based on 14 parameters. Table S6: Confusion matrix from NB classification of 125 colony fingerprints based on 14 parameters. Table S7: Confusion matrix from ANN classification of 125 colony fingerprints based on 14 parameters. Table S8: Confusion matrix from SVM classification of 125 colony fingerprints based on 14 parameters. Table S9: Confusion matrix from RF classification of 125 colony fingerprints based on 14 parameters.
