# Supplementary material for: Colony Fingerprint-Based Discrimination of Staphylococcus species with Machine Learning Approaches
Source: Sensors (Basel). 2018 Aug 24;18(9):2789. doi: 10.3390/s18092789 (PMC6163207; doi:10.3390/s18092789)
Supplement: Supplementary file 1 [file sensors-18-02789-s001.pdf]

Supplementary Information

Table S1. Discriminative parameters used in this study.

Parameter	Abbreviation	Description
Maximal growth rate[a]	μ_{max} (min^{-1})	The maximum value of specific growth rates (μ) after 10 h. The μ was calculated using the following formula: $\mu = \ln(A_t/A_{t-5})/5$, where t is incubation time (every 5 min), and A_t is the area of a colony region after t min of incubation.
Histogram deviation[a]	G	Standard deviation value of the pixel numbers included in 20 bins of the intensity histogram.
Average intensity	I	Average intensity at a colony region
Half central intensity	$I_{1/2}$	Average intensity at a half colony region
Quarter central intensity	$I_{1/4}$	Average intensity at a quarter colony region
Dounutness[a]	D	$I_{1/2}$ divided by I
Central dounutness	D_c	$I_{1/4}$ divided by $I_{1/2}$
Entropy[a]	H	A scalar value representing the randomness of an image [1].
Energy	En	Mean of the amplitude of a fast Fourie transform image, representing the regularity of intensity distribution in a colony region.
Energy density[a]	Ed	Sum of the amplitude of a fast Fourie transform image, representing the regularity of intensity distribution in a colony region.
Weighted center difference	W	The distance (pixel) between the geometric center of a colony region regardless of intensity distribution and the weighted center of the colony region taking intensity distribution into account.
Roundness	R	Eccentricity of a colony region
Zernike moment	Z	A scalar value representing the aberration type of a binary image. In this study, aberration scores with three-dimension and three-item were employed.
Solidity	S	Area of a colony region/area of the convex shell

[a] The parameters used in our previous study [2].

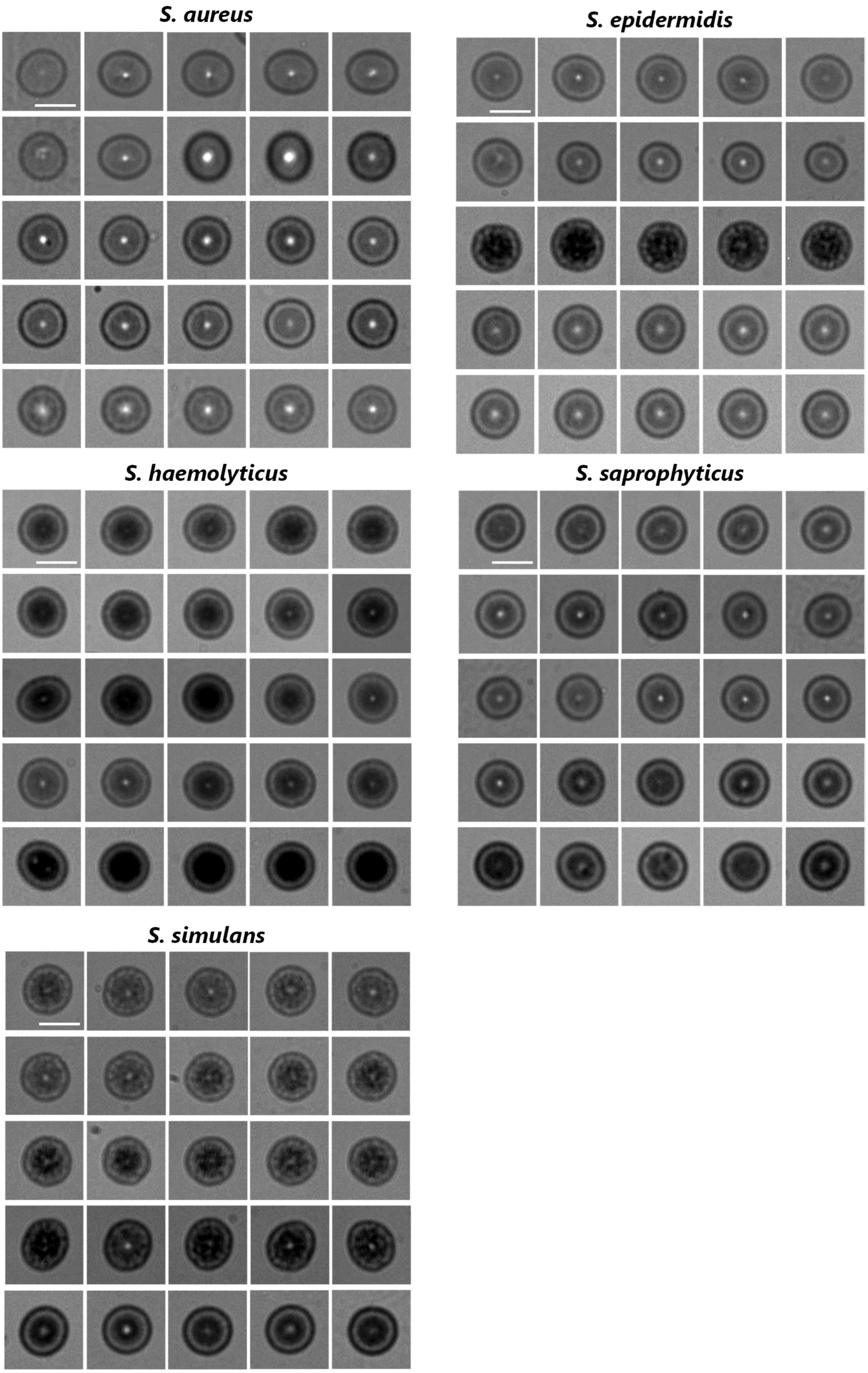

Figure S1. The colony fingerprints of five *Staphylococcus* spp., acquired in this study (scale bar = 200 μm).

Table S2. Confusion matrix from linear discrimination analysis (LDA) classification of 125 colony fingerprints (25 colony fingerprints × 5 *Staphylococcus* spp.) based on five parameters (maximal growth rate (μmax), histogram deviation (G), donutness (D), entropy (H), and energy density (Ed).

prediction \ actual	*S. aureus*	*S. epidermidis*	*S. haemolyticus*	*S. saprophyticus*	*S. simulans*
S. aureus	20	1	0	0	0
S. epidermidis	4	18	0	2	5
S. haemolyticus	0	0	16	0	1
S. saprophyticus	1	1	3	20	0
S. simulans	0	5	6	3	19

The numbers in yellow boxes are the colony number correctly classified.

Table S3. ANOVA for the parameters.

Parameter	F-value	p-value
Dounutness (D)	80.5	4.7×10^{-33}
Half central intensity ($I_{1/2}$)	73.7	2.1×10^{-31}
Entropy (H)	24.2	1.1×10^{-14}
Central dounutness (D_c)	24.1	1.2×10^{-14}
Quarter central intensity ($I_{1/4}$)	20.4	7.3×10^{-13}
Histogram deviation (G)	18.5	7.0×10^{-12}
Maximal growth rate (μ_{max})	17.6	2.3×10^{-11}
Energy (En)	15.8	2.0×10^{-10}
Average intensity (I)	15.4	3.6×10^{-10}
Energy density (Ed)	15.0	5.8×10^{-10}
Zernike moment (Z)	2.4	5.1×10^{-2}
Solidity (S)	2.4	5.5×10^{-2}
Weighted center difference (W)	0.9	4.6×10^{-1}
Roundness (R)	0.3	9.1×10^{-1}

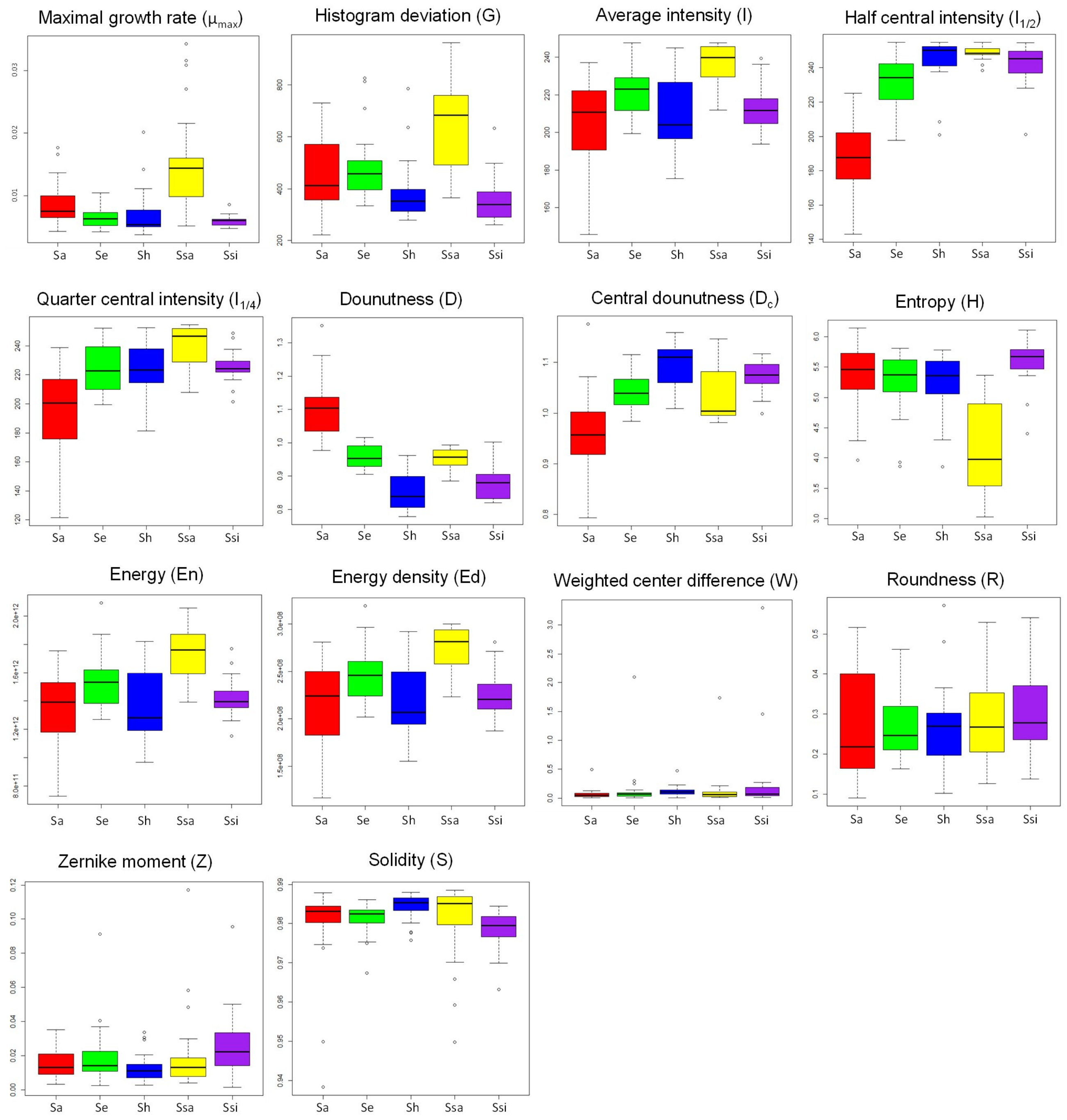

Figure S2. Distribution of the values of the discriminative parameters extracted from the colony fingerprints of 5 *Staphylococcus* spp., i.e., *S. aureus* (Sa, red), *S. epidermidis* (Se, green), *S. haemolyticus* (Sh, blue), *S. saprophyticus* (Ssa, yellow), and *S. simulans* (Ssi, purple).

Table S4. Confusion matrix from LDA classification of 125 colony fingerprints (25 colony fingerprints × 5 *Staphylococcus* spp.) based on 14 parameters (μ_{max}, G, I, $I_{1/2}$, $I_{1/4}$, D, D_c, H, En, Ed, W, R, Z, S).

prediction \ actual	*S. aureus*	*S. epidermidis*	*S. haemolyticus*	*S. saprophyticus*	*S. simulans*
S. aureus	21	1	0	0	0
S. epidermidis	4	19	1	3	6
S. haemolyticus	0	0	19	0	1
S. saprophyticus	0	1	2	22	0
S. simulans	0	4	3	0	18

The numbers in yellow boxes are the colony number correctly classified.

Table S5. Confusion matrix from k-nearest neighbor algorithm (k-NN) classification of 125 colony fingerprints (25 colony fingerprints × 5 *Staphylococcus* spp.) based on 14 parameters (μ_{max}, G, I, $I_{1/2}$, $I_{1/4}$, D, D_c, H, En, Ed, W, R, Z, S).

prediction \ actual	*S. aureus*	*S. epidermidis*	*S. haemolyticus*	*S. saprophyticus*	*S. simulans*
S. aureus	22	0	0	0	0
S. epidermidis	3	21	3	2	6
S. haemolyticus	0	1	19	0	2
S. saprophyticus	0	2	2	22	0
S. simulans	0	1	1	1	17

The numbers in yellow boxes are the colony number correctly classified.

Table S6. Confusion matrix from naive Bayes classifier (NB) classification of 125 colony fingerprints (25 colony fingerprints × 5 *Staphylococcus* spp.) based on 14 parameters (μ_{max}, G, I, $I_{1/2}$, $I_{1/4}$, D, D_c, H, En, Ed, W, R, Z, S).

prediction \ actual	*S. aureus*	*S. epidermidis*	*S. haemolyticus*	*S. saprophyticus*	*S. simulans*
S. aureus	22	0	0	0	0
S. epidermidis	3	21	2	0	4
S. haemolyticus	0	0	19	3	0
S. saprophyticus	0	2	2	22	1
S. simulans	0	2	2	0	20

The numbers in yellow boxes are the colony number correctly classified.

Table S7. Confusion matrix from artificial neural network (ANN) classification of 125 colony fingerprints (25 colony fingerprints × 5 Staphylococcus spp.) based on 14 parameters (μ_{max}, G, I, $I_{1/2}$, $I_{1/4}$, D, D_c, H, En, Ed, W, R, Z, S).

prediction \ actual	*S. aureus*	*S. epidermidis*	*S. haemolyticus*	*S. saprophyticus*	*S. simulans*
S. aureus	25	0	0	0	0
S. epidermidis	0	25	0	0	0
S. haemolyticus	0	0	24	0	0
S. saprophyticus	0	0	1	25	0
S. simulans	0	0	0	0	25

The numbers in yellow boxes are the colony number correctly classified.

Table S8. Confusion matrix from support vector machine (SVM) classification of 125 colony fingerprints (25 colony fingerprints × 5 *Staphylococcus* spp.) based on 14 parameters (μ_{max}, G, I, $I_{1/2}$, $I_{1/4}$, D, D_c, H, En, Ed, W, R, Z, S).

prediction \ actual	*S. aureus*	*S. epidermidis*	*S. haemolyticus*	*S. saprophyticus*	*S. simulans*
S. aureus	25	0	0	0	0
S. epidermidis	0	24	0	0	1
S. haemolyticus	0	0	25	0	0
S. saprophyticus	0	0	0	25	0
S. simulans	0	1	0	0	24

The numbers in yellow boxes are the colony number correctly classified.

Table S9. Confusion matrix from random forest (RF) classification of 125 colony fingerprints (25 colony fingerprints × 5 *Staphylococcus* spp.) based on 14 parameters (μ_{max}, G, I, $I_{1/2}$, $I_{1/4}$, D, D_c, H, En, Ed, W, R, Z, S).

prediction \ actual	*S. aureus*	*S. epidermidis*	*S. haemolyticus*	*S. saprophyticus*	*S. simulans*
S. aureus	25	0	0	0	0
S. epidermidis	0	25	0	0	0
S. haemolyticus	0	0	25	0	0
S. saprophyticus	0	0	0	25	0
S. simulans	0	0	0	0	25

The numbers in yellow boxes are the colony number correctly classified.

References

1. Gonzalez, R. C.; Eddins, S. L. *Digital image processing using MATLAB;* Gatesmark Publishing: New Jersey, NJ, USA, 2003.
2. Maeda, Y.; Dobashi, H.; Sugiyama, Y.; Saeki, T.; Lim, T. K.; Harada, M.; Matsunaga, T.; Yoshino, T.; Tanaka, T. Colony fingerprint for discrimination of microbial species based on lensless imaging of microcolonies. *PLoS One* **2017,** *12*, e0174723.
